# Effect of Shot Noise on Simultaneous Sensing in Frequency Division Multiplexed Diffuse Optical Tomographic Imaging Process

**DOI:** 10.3390/s17122752

**Published:** 2017-11-28

**Authors:** Hansol Jang, Gukbin Lim, Keum-Shik Hong, Jaedu Cho, Gultekin Gulsen, Chang-Seok Kim

**Affiliations:** 1 Department of Cogno-Mechatronics Engineering, Pusan National University, Busan 46241, Korea; hsjang888@pusan.ac.kr (H.J.); gblim@pusan.ac.kr (G.L.); kshong@pusan.ac.kr (K.-S.H.); 2 Tu & Yuen Center for Functional Onco-Imaging, Department of Radiological Sciences, University of California, Irvine, CA 92697, USA; jaeduc1@uci.edu (J.C.); ggulsen@uci.edu (G.G.)

**Keywords:** shot noise, diffuse optical tomography, time-division multiplexing, frequency-division multiplexing

## Abstract

Diffuse optical tomography (DOT) has been studied for use in the detection of breast cancer, cerebral oxygenation, and cognitive brain signals. As optical imaging studies have increased significantly, acquiring imaging data in real time has become increasingly important. We have developed frequency-division multiplexing (FDM) DOT systems to analyze their performance with respect to acquisition time and imaging quality, in comparison with the conventional time-division multiplexing (TDM) DOT. A large tomographic area of a cylindrical phantom 60 mm in diameter could be successfully reconstructed using both TDM DOT and FDM DOT systems. In our experiment with 6 source-detector (S-D) pairs, the TDM DOT and FDM DOT systems required 6.18 and 1 s, respectively, to obtain a single tomographic data set. While the absorption coefficient of the reconstruction image was underestimated in the case of the FDM DOT, we experimentally confirmed that the abnormal region can be clearly distinguished from the background phantom using both methods.

## 1. Introduction

Diffuse optical tomography (DOT) using near-infrared (NIR) light is a promising medical diagnostic imaging technique because of its high imaging contrast performance in distinguishing tumors from normal tissue [1,2]. As the optical absorption spectra of oxy- and deoxy-hemoglobin are distinctly different in the NIR range [3], functional medical information, such as metabolism, blood flow, and oxygen saturation, is recovered by measuring the optical tissue parameters [2,4,5]. A tomographic reconstruction algorithm based on the photon diffusion model allows quantitative depth-resolved information in thick tissue. Owing to these capabilities, applications of DOT include quantitative functional cerebral studies [4,6,7], disease diagnosis [5], and neonatal hemorrhage detection [2,5]. In addition, DOT influences the monitoring response to neoadjuvant and imaging of surgery guidance.

In early DOT systems, the sequential illumination method of time-division multiplexing (TDM), was commonly used for encoding and decoding source-detector (S-D) pairs [8]. Depending on the number of source channels, the TDM method may require a long time to measure a single tomographic data set, which causes a reduction in temporal resolution [8]. Therefore, TDM DOT is not appropriate for applications which require high temporal resolution, such as the monitoring of blood flow, changes oxygen saturation, and response of vasoactive agents [9]. In the last decade, there have been extensive efforts to enhance the temporal resolution of the DOT system. Frequency-division multiplexing (FDM), a method in which multiple sources with different modulated frequencies illuminate the target simultaneously, has been useful in effectively improving temporal resolution [7,10,11,12]. Owing to this advantage, the FDM method has been studied to examine rapid hemodynamic activities which require sufficient temporal resolution, such as cerebral blood flow and blood oxygen saturation [7]. However, the FDM method relies on an array of sources and detectors, and optical signals measured by optical detectors have unacceptable low signal to noise ratios (SNRs) [7]. This is because a strong optical signal from the nearest source channel overwhelms a week optical signal from a farther source channel. The signals which have unacceptably low SNR could lead to an increase in the distortion of the reconstruction images. For this reason, most articles on FDM DOT systems have treated the reconstruction images of the shallow area. To our knowledge, this paper is the first to treat the thick phantom as a cylindrical phantom 60 mm of diameter. We also include a comparison of the recovered images obtained by our FDM DOT system with TDM DOT by considering the temporal resolution and image quality.

According to the temporal characteristics of the considered light sources, DOT can be classified as either continuous wave (CW), frequency-domain (FD), or time-domain (TD) [12]. CW-DOT technology is a powerful method for functional imaging studies because compared with FD-DOT and TD-DOT, and it is able to utilize inexpensive light sources and increase the dynamic range [13,14]. We predict that a quantitative study comparing the TDM and FDM methods of CW-DOT will be helpful in finding conditions that improve temporal resolution without a reduction in image quality. Additionally, a quantitative comparison between the frequency encoded method (FDM) and the sequential illuminating method (TDM) will greatly assist fast diffuse optic imaging studies. Especially, when more than two sources are simultaneously turned on in FDM DOT, shot noise plays an important role in selecting appropriate configuration to receive sufficient signals in relation to the placement of sources and detectors.

In this study, we developed FDM DOT systems to measure a large cylindrical phantom 60 mm in diameter and compared the reconstructed image with that of other images obtained from TDM DOT to evaluate the performances of the two systems. In the case of TDM DOT, a laser diode was used to sequentially illuminate the phantom. In the case of FDM DOT, 6 laser diodes encoded at different modulation frequencies illuminated the phantom simultaneously. We analyzed the data acquisition rates and SNRs of all S-D pairs to compare the performances of TDM DOT and FDM DOT using the temporal resolution and image quality.

## 2. Methods

**Figure 1:**
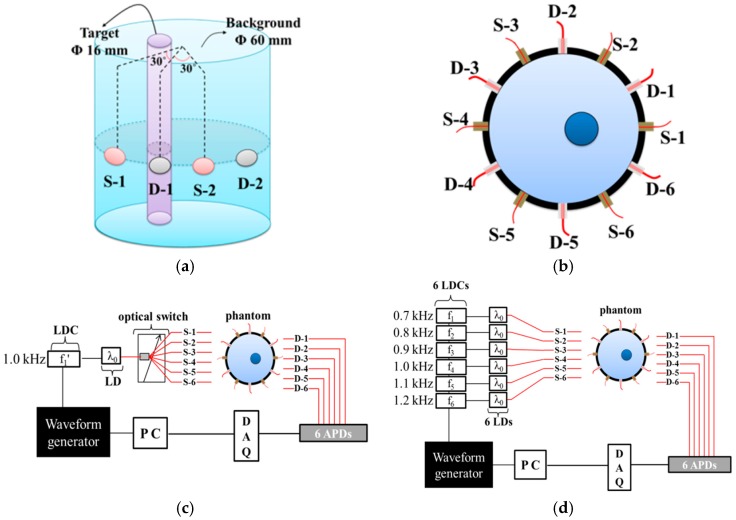
Schematic diagrams of (**a**) the cylindrical phantom and (**b**) the S-D interface configuration, block diagrams of experimental setups for (**c**) the time-division multiplexing (TDM) diffuse optical tomography (DOT) system and (**d**) the frequency-division multiplexing (FDM) DOT system; “LDC”, “LD”, and “DAQ” are acronyms for “laser diode controller”, “laser diode”, and “data acquisition system”, respectively.

For the two-dimensional (2D) imaging reconstruction process in many researches, cylindrical phantom has been widely used to understand the modality of the DOT imaging systems of 2D diffusing space [13,14]. We prepared a large area cylindrical phantom with a diameter of 60 mm to mimic the optical properties of biological tissue. The phantom comprised of agarose gel mixed with intra-lipid and India ink. The background absorption and reduced scattering coefficients of the phantom were 0.005 and 1 mm^−1^, respectively. We inserted a cylindrical target with a diameter of 16 mm, located 7 mm away from the center of the cylindrical phantom, as shown in Figure 1a. The absorption and reduced scattering coefficients of the target were 0.02 and 1 mm^−1^, respectively. The source and detector probes were alternately arranged at the surface of the phantom every 30°, as shown in Figure 1b.

Figure 1c shows the conventional TDM DOT system. In the TDM system, a laser diode with a center wavelength of 780 nm was used as the optical source. A laser diode controller (LDC) provided sinusoidal modulation to the laser diode and the intensity of the laser beam was modulated at a rate of 1 kHz. The laser beam passed through an optical switch (MEMS biomedical 1 × N optical switch, DiCon Fiberoptics, Richmond, CA, USA), and sequentially illuminated each source channel at a switching frequency of 1 Hz. The experimental optical loss of the optical switch was 0.83 dB. The diffused photons in the phantom were collected by fiber-bundle probes and guided directly to avalanche photodiode (APD) modules. In contrast with the source illumination, all of the detectors received the optical signals simultaneously and continuously. The analog electrical signals from the APD modules were digitized at a sampling rate of 10 kHz.

Figure 1d shows the proposed FDM DOT system. In the FDM method, a set of 6 laser diodes was used as an optical source. Using 6 laser diode controllers, the intensities of the 6 laser diodes were modulated at frequencies of 0.7, 0.8, 0.9, 1.0, 1.1, and 1.2 kHz to avoid overlapping of harmonic frequencies. These modulated lasers illuminated the phantom simultaneously. The optical signals from the 6 laser diodes were measured simultaneously, as in the TDM method, by 6 APD modules. In the FDM DOT system, the gain of the APD modules was reduced by 10 dB to prevent detector saturation. The analog electrical signals from the APD modules were converted to digital signals at a sampling rate of 10 kHz.

**Figure 2:**
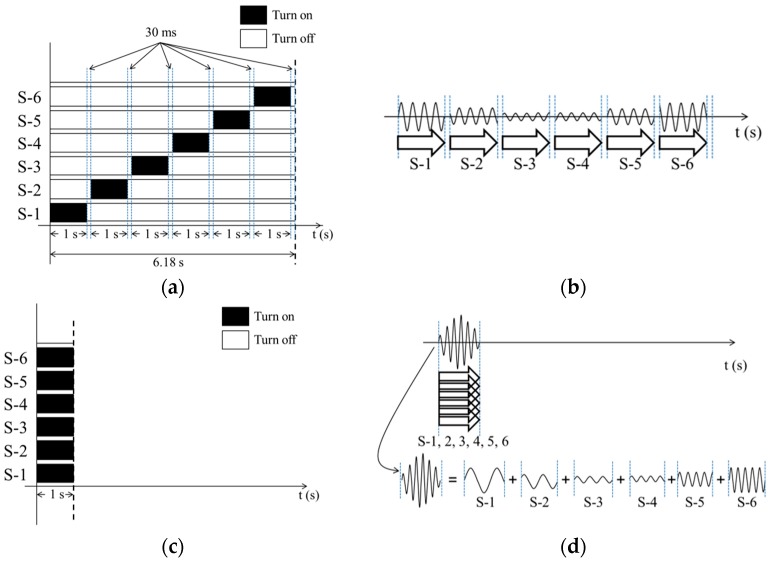
(**a**) Source illumination sequence and (**b**) data acquisition charts of TDM. (**c**) Source illumination sequence and (**d**) data acquisition charts of FDM.

In order to obtain the quantities of light at all S-D pairs, proper de-multiplexing processes are necessary in both the TDM and FDM methods. For an effective explanation of the de-multiplexing process, we present the illumination sequence and data acquisition charts in Figure 2. For TDM DOT, the APD received optical signals from 6 source channels in serial order, as shown in Figure 2a. Therefore, the sequential optical signals should be sorted by time. Considering a data acquisition rate of 10 kHz, the first 10,000 samples of data corresponding to time of 1 s, contained optical signals from the S-1 channel. The next 300 samples corresponding to a switching time of 30 ms, had invalid information. Beyond these 300 samples, the optical signals from the S-2 source channel began and continued during the next 10,000 samples. In the same way, the whole signals from an APD can be divided into de-multiplexed signals, as shown in Figure 2b. Fast Fourier transforms (FFT) were performed on the de-multiplexed signals corresponding to each S-D pair. When we performed the FFT, the window size was 1000 samples with zero padding which produced a FFT size of 1024 samples. The total acquired data size for an S-D pair and window size were 10,000 and 1000 samples, respectively, and ten frames of frequency domains were obtained. The magnitudes of the peaks at 1 kHz in ten frames of the frequency domains were averaged and used as the tomographic data set. For FDM DOT, all of the light sources illuminated the phantom simultaneously for the first 1 s, as shown in Figure 2c. In order to de-multiplex the mixed optical signals in the time domain, FFT was performed in the same way as for TDM DOT. The magnitudes of the peaks at each modulated frequency in the frequency spectra were recorded and an average was obtained over 10 frames of the frequency domain. Even though total signal acquisition time of TDM DOT is 6.15 times longer than FDM DOT as shown in Figure 2, the number of acquired signal data from each source of the TDM and FDM DOT are the same amount of 10,000 samples. For this reason, there were no bandwidth narrowing effects during the FFT process. The tomographic data set was used for the image reconstruction using the tomographic reconstruction software NIRFAST [15,16].

In general, the in vivo imaging system can be affected by various physiological factors, such as from subject cardiac or respiratory cycle. These factors act as noise in the imaging system and reduce the imaging accuracy. Usually, DOT system consists of multiple S-D channels and the physiological noise affects across a number of S-D channels. In addition, systemic noise, such as thermal or electrical noise, can also affect the overall system. Therefore, it is important to identify the noise covariance across S-D channels to figure out characteristics of the DOT system. It has been already well identified that the SNR of the image can be improved by considering the noise variance or covariance matrix during the inverse problem process for in vivo diffuse optical imaging, especially [17]. On the other hand, for the static phantom study, it is also widely allowed to assume the noise per channel as a constant value to consider the noise covariance independently [17,18,19,20]. In this research, the effect of shot noise from other sources is mainly analyzed during the image reconstruction process. Thus, we also follow the general assumption that the noise per channel can be independently identified because the shot noise is determined by the amount of incident light irrespective of the channel. To reconstruct the tomographic image using NIRFAST software, the data for the phantom consisting only of the background was first obtained to normalize the intensity of the detected light and to solve the inverse problem. Next, the data for the inhomogeneous phantom shown in Figure 1a was acquired. Finally, the data of the homogeneous phantom was normalized and compared with simulated data in the NIRFAST software.

## 3. Results and Discussion

To analyze the temporal resolutions of the TDM DOT and FDM systems, we measured the operation time for a single tomographic data set. Table 1 shows operation times of the TDM and FDM systems during obtaining a single tomographic data set. The total measurement times of the TDM and FDM systems were 6.18 and 1 s, respectively. Generally speaking, in TDM DOT, the total operation time obtained for a single data set can be expressed as *n*(*T_DAQ_* + *T_SW_*), where *n* is the number of light sources, *T_DAQ_* is the data acquisition time for each source channel, and *T_SW_* is the channel switching time of the optical switch. In the case of FDM DOT, the operation time can be expressed as *T_DAQ_*. In our experiment, *T_DAQ_* and *T_s_* were 1 s and 30 ms, respectively. Therefore, the temporal resolution of the FDM system was *n*(*T_DAQ_* + *T_SW_*)/*T_DAQ_* times faster than that of the TDM system.

In order to impartially compare TDM DOT with FDM DOT, the total data acquisition times should be the same. Though TDM DOT has a longer operation time, the data acquisition occurred during the entire operation time. In the case of the TDM DOT system, the data acquisition times of all S-D pairs were the same as *T_DAQ_* and the data at each S-D pair were measured at different times according to the source channel. The total data acquisition times were the same as *T_DAQ_* in both the TDM DOT and FDM DOT systems.

**Figure 3:**
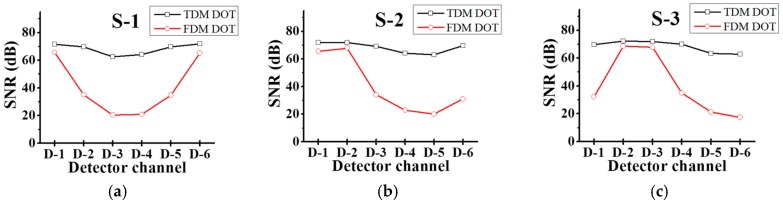

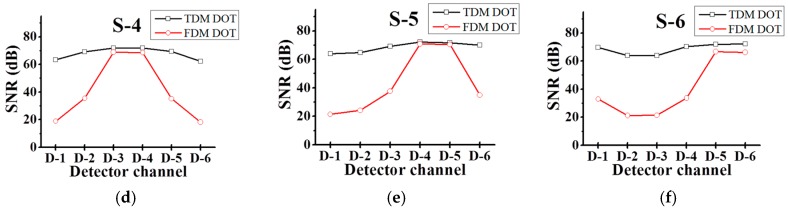
Graphs of signal to noise ratio (SNR) versus S-D pairs at (**a**) Source 1, (**b**) Source 2, (**c**) Source 3, (**d**) Source 4, (**e**) Source 5, and (**f**) Source 6. The black and red solid lines with circles represent the TDM DOT and FDM DOT systems, respectively.

The SNRs of the two systems were compared in Figure 3 to analyze the impact of SNR on the measured optical signals on image quality. Figure 3 shows the SNR of each source (1 to 6) and detector (1 to 6) pair. As clearly shown, the SNR of the farthest S-D pair was generally lower than that of the relatively near one in both systems. This is because the optical signals in each case of the farther S-D pair were weaker than those in the case of the nearer ones.

To investigate further, we analyzed the SNR of each system in detail. In the FDM system, the total noise level was increased owing to the additional shot noise generated by the other source channels. The shot noise generated in the detectors can be explained using the uncertainty principle [21]. The magnitude of the shot noise is proportional to the square root of the number of photons captured by the detectors [22] and can therefore be described by Equations (1) and (2) for the TDM and FDM systems, respectively.

(1) $$N_{shot\;noise}\;\sim \;\sqrt{N_{S-1}}$$

(2) $$N_{shot\;noise}\;\sim \;\sqrt{N_{S-1}+N_{S-2}+N_{S-3}+N_{S-4}+N_{S-5}+N_{S-6}}$$

The SNR affected by the shot noise (for example, from source 1) can then be described by Equations (3) and (4) for the TDM and FDM systems, respectively.

(3) $$SNR_{S-1}\;\sim \;\frac{N_{S-1}}{\sqrt{N_{S-1}}}=\sqrt{N_{S-1}}$$

(4) $$N_{shot\;noise}\;\sim \;\frac{N_{S-1}}{\sqrt{N_{S-1}+N_{S-2}+N_{S-3}+N_{S-4}+N_{S-5}+N_{S-6}}}$$

The SNR in the TDM system depends on only one of the optical sources, whereas in the FDM system, it depends on all of the optical sources. Therefore, in the FDM system, the SNR was significantly reduced when the distances between the S-D pairs was large. These equations indicate that the SNR in the FDM system was lower than that in the TDM system, confirming the experimental results in Figure 3.

**Figure 4:**
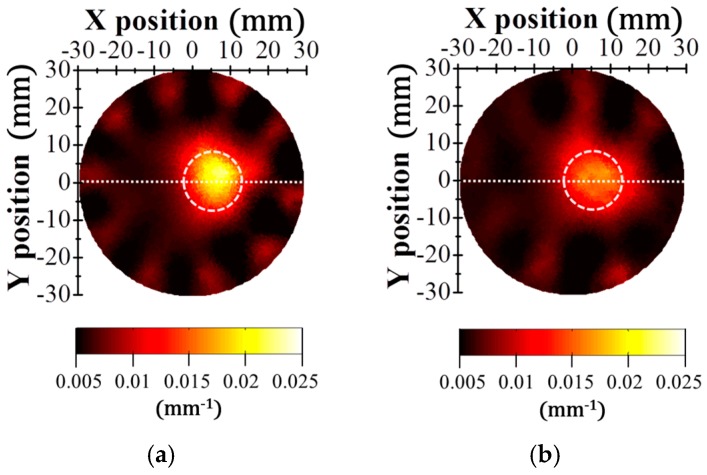
(**a**) Reconstructed tomographic images of the phantom absorption coefficients obtained by the TDM DOT and (**b**) FDM DOT systems.

**Figure 5:**
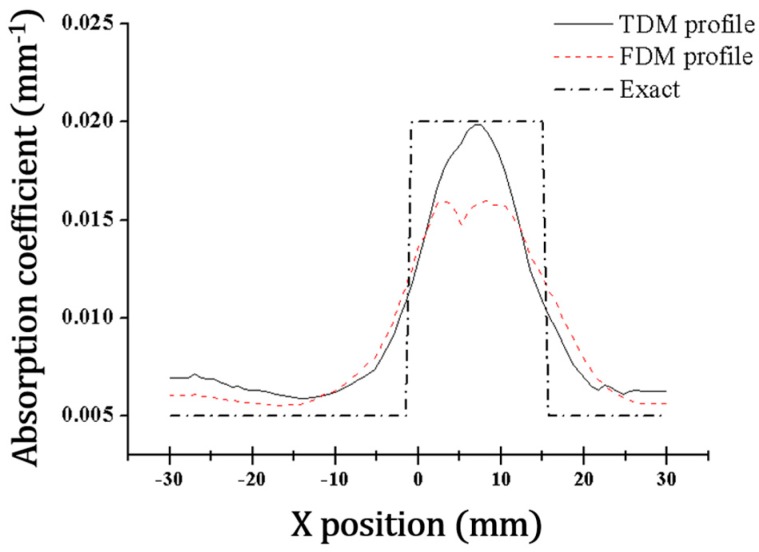
Cross sectional profiles of the phantom absorption coefficients along horizontal dotted lines in Figure 4. The black dash-dot line represents the actual phantom absorption coefficient profile. The black solid and red dashed lines represent the estimated absorption coefficient profiles recovered by the TDM DOT and FDM DOT systems, respectively.

The SNR of an optical signal affects the quality of a reconstructed image. The data sets were reconstructed into DOT images using the NIRFAST software program. Figure 4 shows the reconstructed absorption coefficient maps of the phantom. The reconstruction images were obtained with obvious contrast between the background and target in both the TDM DOT and FDM DOT systems. However, the contrast of the image obtained by the TDM system is clearer than that obtained by the FDM system owing to the high SNR of the TDM system. In the reconstructed images, we inserted white dotted lines to indicate the actual abnormal regions. For both systems, the location of the target was also clearly recovered compared with the exact position. For a more detailed analysis of the absorption coefficient map, we examined the absorption coefficient profiles along the horizontal dotted line shown in Figure 4. Figure 5 shows the cross-sectional absorption coefficient profiles obtained by the TDM and FDM systems. The recovered absorption coefficients were 0.020 and 0.016 mm^−1^ in the TDM and FDM systems, respectively. Based on our measurements, the estimation of the absorption coefficient in the TDM system was more precise than that in the FDM system. The estimation error of the FDM system was largely due to the S-D pairs that were the farthest apart, which had the lowest SNR values.

In order to improve the quality of the DOT image, the number of source and detector channels is normally increased for TDM DOT. However, this is not always true for FDM DOT. If we increased the number of source and detector channels for the same phantom size, the sources and detectors would be arranged more closely. In other words, while the distances between the farthest S-D pairs would still be similar or unchangeable, the distances between the nearest S-D pairs would gradually reduce due to the increased number of source and detector channels. Reducing the distance between the nearest S-D pair leads to an increase in the magnitude of the shot noise generated by the nearest sources. In this situation, optical signals from the farthest source would be largely influenced by the shot noise from the nearest source. As a result, the SNRs of the farthest S-D pairs would decrease and the image quality would depreciate under a certain SNR.

**Figure 6:**
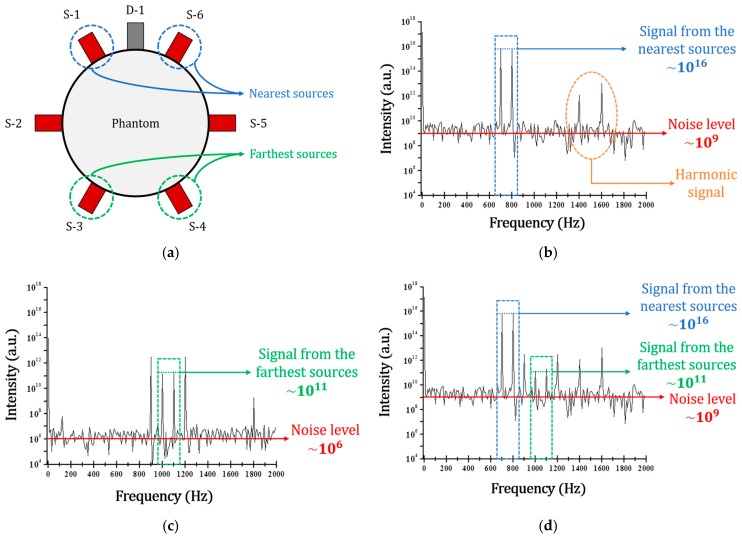
(**a**) Schematic setup of experiment, (**b**) FFT result when turned on the only the nearest two sources of S-1 and S-6, (**c**) FFT result when turned on far four sources of S-2, S-3, S-4, and S-5, (**d**) FFT result when turned on all of 6 sources.

Figure 6 shows the experimental setup and the results to confirm the effect of shot noise on the FFT process. Figure 6a represents that a single detector D-1 only is analyzed from the 6 S-D pairs configuration of Figure 1b. As shown in Figure 6a, 6 sources (S-1, S-2, S-3, S-4, S-5, and S-6) are arranged counterclockwise with respect to the detector D-1, and they are modulated at different frequencies from 700 to 1200 Hz. Figure 6b shows the FFT results of FD DOT data acquired with only the nearest two sources (S-1 and S-6) turned on. As can be seen in Figure 6b, the signal intensity obtained from the nearest sources are very high with ~10^16^. Noise level shown in Figure 6b is also high with ~10^9^ due to the huge photon flux from the nearest sources. Therefore, the SNR of the nearest sources, S-1 and S-6, show approximately 70 dB. Figure 6c shows the FFT results of FD DOT data acquired with four sources (S-2, S-3, S-4, and S-5) turned on without the nearest sources (S-1 and S-6). As can be seen from Figure 6c, signal from the farthest sources (S-3 and S-4) are significantly weaker than the signals from the nearest sources (S-1 and S-6) of Figure 6b. However, due to the low light level, shot noise is significantly reduced to ~10^6^ and its SNR with the farthest sources is calculated to approximately 50 dB. Figure 6d shows the FFT results of FD DOT data acquired with all 6 sources turned on. As shown in Figure 6d, signal levels of the nearest and the farthest sources are same with the results of Figure 6b,c, respectively. However, shot noise is significantly increased due to the strong photon flux from the nearest sources. Therefore, the SNR with the farthest sources (S-3 and S-4) significantly drop to 20 dB, while the SNR of the nearest sources (S-1 and S-6) are still 70 dB due to the main influence of shot noise from the nearest sources. Because of the reduced SNR, the intensity of the farthest sources become more unstable and the accuracy of the measured intensities was also decreased. Thus, the reconstructed image of FD DOT is distorted more due to the reduced accuracy, as shown in Figure 4b and Figure 5.

## 4. Conclusions

We developed FDM DOT systems based on CW-DOT and compared their performances with the TDM DOT system in terms of temporal resolution and image quality. The focus of this study is the analysis of the imaging distortion problems due to the influence of shot noise caused by another source while more than two sources are simultaneously turned on. The results showed that the use of simultaneous illumination sources yielded a faster operation time in the FDM DOT system. For the image quality evaluation using a phantom of 60 mm in diameter, the TDM DOT system was able to successfully recover the target absorption coefficients of 0.02 mm^−1^, while the FDM DOT system was able to recover absorption coefficients of 0.016 mm^−1^ with a 20% error. In the FDM system, the absorption coefficients of the phantom were underestimated in comparison with the actual absorption coefficients. This was attributed to the data sets corresponding to the S-D pairs that were the farthest apart in the FDM DOT system. The considerable quantity of shot noise in the signals affected the reconstruction results. These results will improve our understanding of optimal hardware configurations for a variety of CW-DOT applications. As a result, the TDM DOT has a relatively slow sampling rate but is less influenced by the shot noise. It means that TDM DOT is suitable to measure large size samples and static targets, such as breast cancer imaging. On the other hand, in the case of FDM DOT, it is suitable for fast time varying physiological target imaging, such as functional brain imaging, due to a fast sampling rate. In further studies, it will be necessary to investigate the optimal hardware configuration for three-dimensional (3D) image reconstruction to get more practical biomedical analysis.

## Figures and Tables

**Table 1:** Comparison between the TDM DOT and FDM DOT systems. “*T_DAQ_*” and “*T_SW_*” are acronyms for “data acquisition time” and “switching time”, respectively.

Methods	TDM	FDM
Data acquisition time (*T_DAQ_*)	1 s	1 s
Total operation time (s)	6.18 s (6*T_DAQ_* + 6*T_SW_*)	1 s (*T_DAQ_*)
The number of average for	10	10
Modulated frequencies at each source channel	*f*_1–6_ = 1 kHz	*f*_1_ = 0.7 kHz
		*f*_2_ = 0.8 kHz
		*f*_3_ = 0.9 kHz
		*f*_4_ = 1.0 kHz
		*f*_5_ = 1.1 kHz
		*f*_6_ = 1.2 kHz
Frequency bandwidth (*Δf*)	*Δf*_1–6_ = 10 Hz	*Δf*_1–6_ = 10 Hz
